# Prevalence of Disease and Relationships between Laboratory Phenotype and Bleeding Severity in Platelet Primary Secretion Defects

**DOI:** 10.1371/journal.pone.0060396

**Published:** 2013-04-02

**Authors:** Luca A. Lotta, Alberto Maino, Giacomo Tuana, Raffaella Rossio, Anna Lecchi, Andrea Artoni, Flora Peyvandi

**Affiliations:** 1 Angelo Bianchi Bonomi Hemophilia and Thrombosis Center, Fondazione Istituto Di Ricovero e Cura a Carattere Scientifico Ca′ Granda-Ospedale Maggiore Policlinico, Università degli Studi di Milano and Fondazione Luigi Villa, Milan, Italy; 2 Hematology 1 CTMO, Fondazione Istituto Di Ricovero e Cura a Carattere Scientifico Ca′ Granda-Ospedale Maggiore Policlinico, Milano, Italy; University of Leuven, Belgium

## Abstract

**Background:**

The prevalence of platelet primary secretion defects (PSD) among patients with bleeding diathesis is unknown. Moreover, there is paucity of data on the determinants of bleeding severity in PSD patients.

**Objective:**

To determine the prevalence of PSD in patients with clinical bleeding and to study the relationships between the type of platelet defect and bleeding severity.

**Methods:**

Data on patients referred for bleeding to the Angelo Bianchi Bonomi Hemophilia and Thrombosis Center, Milan (Italy) in the years between 2008 and 2012 were retrieved to study the prevalence of PSD. Demographic, clinical and laboratory information on 32 patients with a diagnosis of PSD was used to compare patients with or without associated medical conditions and to investigate whether or not the type and extension of platelet defects were associated with the bleeding severity score (crude and age-normalized) or with the age at first bleeding requiring medical attention.

**Results:**

The estimated prevalence of PSD among 207 patients with bleeding diathesis and bleeding severity score above 4 was 18.8% (95% confidence interval [CI]: 14.1–24.7%). Patients without associated medical conditions had earlier age of first bleeding (18 vs 45 years; difference: -27 years; 95% CI: -46 to -9 years) and different platelet functional defect patterns (Fisher's exact test of the distribution of patterns, P = 0.007) than patients with accompanying medical conditions. The type and extension of platelet defect was not associated with the severity of bleeding.

**Conclusions:**

PSD is found in approximately one fifth of patients with clinical bleeding. In patients with PSD, the type and extension of laboratory defect was not associated with bleeding severity.

## Introduction

Platelet primary secretion defects (PSD) are defined by reduced primary platelet granule secretion upon stimulation by different platelet aggregation agonists [1]. PSD often results in bleeding tendency, which is usually mild to moderate albeit asymptomatic patients have been described [2]–[4]. The type of laboratory defect is heterogeneous, consisting of reduced aggregation upon stimulation by one single or multiple agonists and reduced response only to low or also to high concentrations of the agonists [5]. PSD may present as an isolated condition or in association with medical conditions or diseases such as autoimmune disorders [6], [7], liver disease [8] or cancer [9]. Systematic data on the prevalence, clinical and laboratory characteristics and determinants of bleeding severity of PSD are scanty. Studies on these defects traditionally presented one or few well characterized patients, perhaps because diagnosing and characterizing PSD requires labor-intensive laboratory testing and the availability of fresh samples. More recently, Quiroga et al. investigated the prevalence of PSD and other hemostatic abnormalities in a cohort of 280 patients referred for mucocutaneous bleeding, yielding a prevalence of approximately 19% for PSD [10]. An even higher percentage of primary secretion defects was found in women with menorrhagia by Philipp et al, but no distinction regarding nature and type of the defects was made [11]. The prevalence of PSD in patients with any type of bleeding and the determinants of bleeding severity within PSD remain unknown.

With this as a background, we collected data on patients recently referred to our institution for bleeding diathesis. We used collected information to study (a) the prevalence of PSD in patients with bleeding, (b) the demographic, clinical and laboratory differences between PSD patients with or without accompanying medical conditions, and (c) the relationships between platelet testing results and bleeding severity in patients with PSD.

## Methods

### Patients

Patients with bleeding or hemostatic testing abnormalities are referred to the general hematology or to the von Willebrand disease/rare bleeding disorder outpatient clinics of the Angelo Bianchi Bonomi Hemophilia and Thrombosis Center, Milan (Italy) where they undergo a first clinical visit with collection of detailed medical history (including pharmacological anamnesis, individual and familial history of bleeding and bleeding severity score [BSS] compilation as described by Tosetto et al. [12], [13]). A copy of the questionnaire used to compile BSS is in Table S1. Patients also undergo blood collection for first level diagnostic tests, which include complete blood count, measurement of prothrombin time, activated thromboplastin time, von Willebrand factor (VWF) antigen, and VWF ristocetin cofactor activity [14]. Patient with elevated BSS (i.e. a score of 4 or more) and normal testing are then referred to the platelet disorder clinic for platelet functional testing.

For this study, patients seen at the general hematology and at the von Willebrand and rare-bleeding disorder out-patient clinics of the Hemophilia and Thrombosis Center in the time period between January 2008 and March 2012 were screened for inclusion. Patients were included in the study if they matched the following criteria: (a) they were referred for diagnostic workup following a bleeding episode or for hemostatic testing abnormalities; (b) at their first visit to the center they were found to have clinical history of bleeding, with a BSS equal or above 4. The rationale of using a cutoff of BSS of 4 or above to define abnormal bleeding tendency relies on the results of a recent study in 100 apparently healthy individuals, showing that the upper bound of BSS range in the normal population is 3.6 [15]. Therefore, patients with a score of 4 or more were deemed to have abnormal bleeding history.

### Definition of PSD and platelet functional testing

Patients were tested for PSD when they had normal platelet counts at the time of first visit, they were found to have normal VWF antigen and ristocetin cofactor activity, and they had normal prothrombin and activated thromboplastin times.

To characterize platelet function, patients underwent the following examinations: (a) measurement of platelet GpIb/IX/V and GpIIb/IIIa surface expression, (b) testing of platelet granule-content secretion upon stimulation by different agonists and (c) platelet granule content measurement. PSD was defined by (a) reduced primary platelet granule secretion upon stimulation by at least one of different platelet aggregation agonists (ADP, collagen, U46619 and TRAP); (b) normal surface expression of GpIb/IX/V and and GpIIb/IIIa and (c) normal platelet granule content (serotonin, ATP, ADP, fibrinogen). Examinations were performed on fresh samples on the same day of collection and a negative control (i.e. a friend or non-consanguineous relative of the patient, with no bleeding history, who accompanied the patient to the hospital and agreed to be tested) was tested in parallel with patient samples in each experiment. Platelet secretion was defined defective when (a) testing results were below a normal range established by secretion in up to 96 controls with no bleeding history and (b) were below the levels measured for the control sample that was tested with patient samples on the day of examination. Patients were not tested for platelet secretion when they were actively taking medications that may affect the results of secretion testing; in this case, patients were requested to withdraw medications and were tested after a washout period. Drugs that were paid particular attention to were non-steroidal anti-inflammatory drugs, antiplatelet agents and serotonin reuptake inhibitors. Blood samples were collected in 0.129 mol/L sodium citrate and centrifuged at 150 g for 15 minutes to obtain platelet rich plasma, which was used for the tests. Measurement of platelet GpIb/IX/V and GpIIb/IIIa expression was performed by flow cytometry as previously described [16]. Platelet secretion was assessed by incubating samples of platelet rich plasma (0.45 mL) with 50 µL of luciferin/luciferase reagent at 37°C for 30 seconds and stirring at 1000 rpm in a lumiaggregometer (Lumi-aggrometer, Chrono-log Corp). After incubation, 10 µL of one of the agonist agents was added and ATP secretion and aggregation tracings were recorded for 3 minutes [17]. Employed agonists were adenosine diphosphate (ADP, Sigma-Aldrich Co., St. Louis, USA) at 4 and 20 µM final concentrations, collagen (Mascia Brunelli, Milano, Italy) at 2, 4 and 20 µg/mL final concentrations, thrombin receptor-activating peptide (TRAP, Sigma-Aldrich Co., St. Louis, USA) at 10 and 20 µM final concentrations and the thromboxane A2 analogue, U46619 (Sigma-Aldrich Co., St. Louis, USA), at 0.5 and 1 µM final concentrations. Normal ranges (2.5th and the 97.5th percentiles of the distribution in controls) of platelet secretion testing results were as follows (all expressed in nmol of ATP/10^8^ platelets): ADP 4 µM, 0.022–0.982 (number of controls tested to establish range, n = 96); ADP 20 µM, 0.036–0.612 (n = 59); collagen 2 µg/mL, 0.168–0.932 (n = 62); collagen 4 µg/mL, 0.216–1.260 (n = 68); collagen 20 µg/mL, 0.267–1.475 (n = 55); U46619 0.5 µM, 0.018–1.270 (n = 72); U46619 1 µM, 0.100–1.030 (n = 55); TRAP 10 µM, 0.012–1.074 (n = 41); TRAP 20 µM, 0.094–1.419 (n = 26). Measurement of intra-platelet granule content was performed by the orto-phtaldialdehyde method for serotonin, by the luciferine-luciferase method for ADP and ATP, and by ELISA for fibrinogen [18]. The normal range of values of platelet granule content was calculated by measuring platelet content in 34 controls. Ranges (2.5th and the 97.5th percentiles of the distribution in controls) were as follows: serotonin, 0.19–0.40 nmol/10^8^ platelets; ADP, 1.30–2.88 nmol/10^8^ platelets; ATP, 3.17–7.07 nmol/10^8^ platelets; ATP/ADP ratio, 1.55–3.42; fibrinogen, 0.03–0.19 mg/10^9^ platelets. All patients included in the study and diagnosed with PSD tested within the normal range for any of the aforementioned measurements of platelet content.

### Definition of clinical variables

Data on PSD patients were collected from digital and hard copy clinical records. Information was retrieved on the following: age at referral (i.e. age at the first clinical visit), age of the first bleeding requiring medical attention, sex, region of residence, prevalence of mucocutaneous bleeding symptoms (i.e. cutaneous bleeding, epistaxis, bleeding from minor wounds or oral cavity bleeding), bleeding following surgery, tooth extraction or peri-partum hemorrhage, vaginal bleeding, muscular hematomas, hemarthrosis or intracranial bleeding, BSS, age-normalized BSS (calculated dividing BSS by the age at referral), presence of deficient secretion upon stimulation by ADP, collagen, U46619 or TRAP at any concentration or at the maximal dose, number of agonists eliciting reduced response at any concentration or at maximal stimulation, dose and pattern of deficient platelet secretion (i.e. the combination of agonists eliciting reduced response upon stimulation). Information on the presence of medical conditions previously reported to be associated with PSD (reviewed by Fuse [1]) was retrieved as well.

### Study of the prevalence

Prevalence was calculated as the proportion of patient with PSD on the total of patients belonging to the source population. The source population was defined as the group of patients (a) referred for diagnostic workup following a bleeding episode or for hemostatic testing abnormalities in the same time period used for patient inclusion (January 2008 and July 2011) and (b) found to have clinical history of bleeding, with a BSS equal or above 4 at clinical interview. While for analyses comparing patients with/without associated conditions and those testing the association of laboratory results with disease severity, we considered all PSD patients recruited from January 2008 and March 2012 (n = 32), for all the analyses investigating prevalence, we only considered PSD patients recruited from January 2008 and July 2011 (n = 27). Patients visited for the first time after July 2011 were excluded from prevalence calculation. We indeed considered that many of these patients still had to complete the diagnostic workup, hence biasing the calculation of the prevalence of PSD towards an underestimation.

In the group of patients who had BSS of 4 or more and were not referred for platelet testing, prevalence was estimated using the multiple imputation method. First, we constructed a logistic regression model using data from patients with known PSD status, with PSD status as dependent variable and age, sex and BSS score as determinants. We obtained an equation of the probability of PSD: log(y)  =  constant + age*Beta_age_ + sex*Beta_sex_ + BSS*Beta_BSS_. We used the equation to calculate the probability of PSD for each of the untested patients given their age, sex and BSS. We calculated the estimated prevalence of the untested group as the mean of all the individual probabilities. The final prevalence estimation in the entire group of patients with bleeding history and BSS of 4 or more was calculated as the weighted average of prevalence estimations in the groups of patients who were tested and that of patients who were not tested for platelet function (i.e. imputed prevalence). Prevalence calculation was performed before and after the exclusion of patients who only had ADP-induced secretion defect (see main text). This was done in order to take into account the possibility that secretion defect exclusive to the ADP pathway may not be specific enough to define PSD.

### Statistical analysis

Continuous variables were summarized by median value and interquartile range (IQR), categorical values by percentages. Prevalence was calculated as the proportion of patient with PSD on the total of patients belonging to the source population defined with the aforementioned criteria. The 95% confidence interval of the prevalence was calculated according to Agresti-Coull [19]. The characteristics of groups of PSD patients with or without accompanying clinical conditions were compared by calculating differences in medians and proportions and computing their 95% CI. Comparisons of non-dichotomous categorical variables were carried out by Fisher's exact test. Linear regression was used to study the association between the number of agonists eliciting reduced secretion and BSS, age-normalized BSS and age of first bleeding requiring medical attention. The association between laboratory results and clinical severity of PSD was assessed before and after the exclusion of patients who only had ADP-induced secretion defect (see above the rationale for this analysis). Kruskal-Wallis test was used to study the aforementioned proxies of bleeding severity across patients with different patterns of platelet defect.

### Ethics statement

The investigations were conducted in accordance with the principle of the declaration of Helsinki. The study was approved by the institutional review board of the Fondazione IRCCS Ca′ Granda – Ospedale Maggiore Policlinico (deliberation of February 21^st^, 2012) and participants gave their written informed consent.

## Results

### Patient characteristics and prevalence of disease

In the analyzed time period, 32 patients were diagnosed with PSD. The characteristics of the patients included in the study are presented in Table 1. Patients were more frequently of female sex, had their first bleeding episode requiring medical attention during young adulthood and had mild to moderate bleeding tendency, as measured by BSS (Table 1). Reduced secretion upon stimulation by ADP was the most frequent laboratory defect and defective response to multiple agonists was a common occurrence (Table 1). Of the 32 patients, 22 had no accompanying medical condition, whereas for 10 patients PSD was associated with one or more disease/medical condition. Associated medical conditions were hepatitis C virus infection (with or without recent liver transplantation surgery), autoimmune disease (a clinical history of immune thrombocytopenic purpura with currently normal platelet counts and rheumatoid arthritis) or neoplasm (myelodysplasia, Hodgkin's lymphoma, colorectal adenocarcinoma, urothelial carcinoma and mammary sarcoma). Bleeding symptoms mainly consisted of mucocutaneous bleeding (n = 29; 91%) or bleeding following surgery, invasive medical procedures or delivery (n = 23; 72%). Menometrorrhagia was frequent and occurred in two thirds of the 24 women (n = 16; 67%). Other spontaneous bleeding symptoms, like muscle hemoatomas or hemarthrosis were rare (both occurred in 2 patients, 6%). None of the patients had intracranial bleeding.

**Table 1:** Demographic, clinical and laboratory characteristics in 32 patients with primary secretion defects.

Variable	Value
Median age at referral, y (IQR)	35 (21–52)
Median age at first bleeding requiring medical attention, y (IQR)	28 (15–42)
Female sex, n (%)	24 (75)
Median bleeding severity score, points (IQR)	6.5 (5–10)
Median age-adjusted bleeding score, points/y (IQR)	0.17 (0.13–0.35)
Secretion defect upon stimulation^a^	
ADP any concentration, n (%)	32 (100)
ADP 20 µM, n (%)	24 (75)
Collagen any concentration, n (%)	13 (41)
Collagen 20 µg/mL, n (%)	1 (3)
U46619 any concentration, n (%)	16 (50)
U46619 1 µM, n (%)	10 (31)
TRAP any concentration, n (%)	12 (38)
TRAP 20 µM, n (%)	3 (9)
Number of agonists with reduced response, n (%)	
1 agonist	8 (25)
2 agonists	9 (28)
3 agonists	13 (41)
4 agonists	2 (6)
Number of agonists with reduced response at maximal stimulation, n (%)	
0 agonists	7 (22)
1 agonist	13 (41)
2 agonists	11 (34)
3 agonists	1 (3)
Pattern of platelet defect, n (%)	
ADP	8 (25)
ADP, TRAP	2 (6)
ADP, U46619	6 (19)
ADP, U46619, TRAP	3 (9)
ADP, collagen	1 (3)
ADP, collagen, TRAP	5 (16)
ADP, collagen, U46619	5 (16)
ADP, collagen, U46619, TRAP	2 (6)

a Number and percentage of patients showing reduced ATP secretion upon stimulation by the reported agonist at the reported concentration.

IQR, interquartile range; ADP, adenosine diphosphate; TRAP, thrombin receptor-activating peptide.

**Figure 1:**
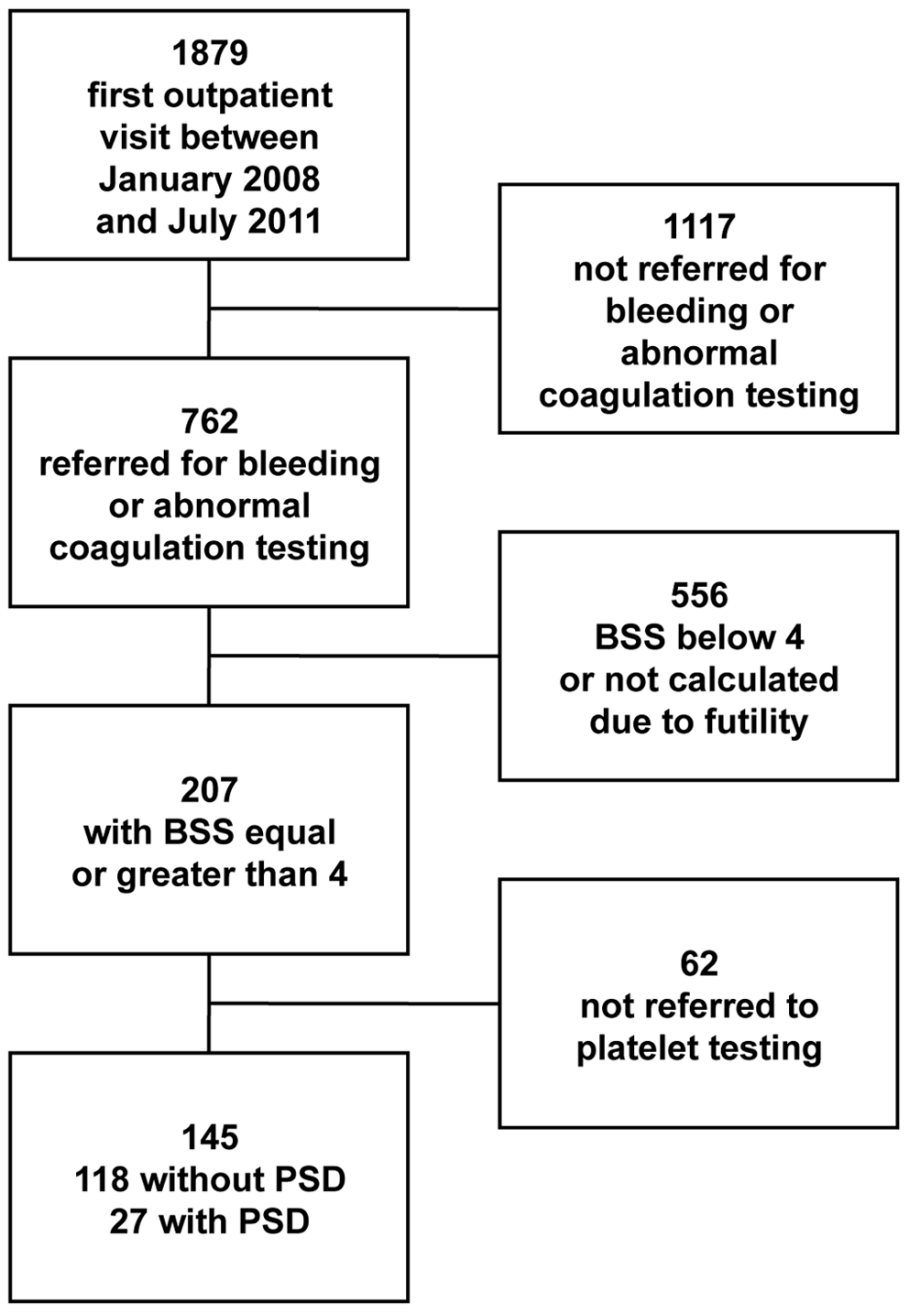
Flow-chart of the study of the prevalence of PSD.

Of the 32 patients, 27 had their first visit in the period between January 2008 and July 2011, so that they were used for the calculation of the prevalence of PSD. Patients visited for the first time after July 2011 (n = 5) were excluded from prevalence calculation (see Methods section “Study of prevalence”). The workflow used for the calculation of prevalence is presented in Figure 1. The prevalence of different diagnoses in 207 patients with bleeding or abnormal coagulation and BSS above 4 is presented in Table 2. In 145 patients who underwent diagnostic screening (see Figure 1), the prevalence of PSD was 18.6%. Multiple imputation in the 62 patients who were not tested for platelet function yielded an estimated PSD prevalence of 19.3%. The weighted mean of the two prevalences yielded a global prevalence in the entire population with bleeding and BBS of 4 or more of 18.8% (95% CI: 14.1–24.7%). Analysis of prevalence was repeated after exclusion of patients with defects only upon stimulation with ADP (see Methods section “Study of prevalence”). This calculation yielded a prevalence of PSD with defects to multiple platelet aggregation agonists of 13.5% (95% CI: 9.6–21.2%). Details on the analysis are provided in Table S2.

**Table 2:** Diagnosis and bleeding severity score values in 207 patients.

Diagnosis	N (%)	Age at diagnosis, median (IQR)	BSS, median (IQR)
Coagulation factor deficiency^a^	27 (13)	37 (15–55)	6 (4–8)
von Willebrand disease	25 (12)	36 (22–4)	9 (6–12)
Primary secretion defects	27 (13)	35 (20–54)	6 (5–10)
Other platelet defect^b^	7 (3)	43 (32–54)	10 (4–15)
Defect in fibrinolysis	2 (1)	29, 38^d^	9, 10^d^
Secondary^c^	8 (4)	56 (35–74)	7 (5–10)
Negative screening			
Including platelet functional testing	49 (24)	41 (26–50)	6 (5–8)
Not tested for platelet function	62 (30)	41 (26–60)	5 (4–6)

Patients with clinical bleeding or coagulation abnormalities and bleeding severity score of 4 or more are presented.

a Including hemophilia A and B or rare bleeding disorders.

b Includes δ-storage pool deficiency and Glanzmanńs thrombasthenia.

c Secondary to drugs or to underlying medical conditions.

d Individual values are reported.

BSS, bleeding severity score.

### Comparison of patients with or without associated medical conditions

The characteristics of the 22 patients without associated medical conditions and those of the 10 patients with associated medical conditions are presented in Table S3. Patients without associated conditions displayed younger age at first bleeding requiring medical attention (patients without vs with associated conditions, median age: 18 vs 45 years, difference: -27 years, 95% CI: -46 to -9 years) and at study enrollment (median age: 34 vs 50 years, difference: -16 years, 95% CI: -34 to 1 years). The distribution of the pattern of platelet defect (i.e. the type of agonist eliciting reduced secretion) was different in the two groups (Fisher's exact test, P = 0.007). Single agonist defect was common in patients without associated conditions, whereas patients with associated conditions always had combined defects (prevalence of single-agonist defect in patients without vs with associated conditions, 36 vs 0%, difference 36%, 95% CI: 4 to 57%).

### Relationships between platelet testing results and bleeding severity

**Figure 2:**
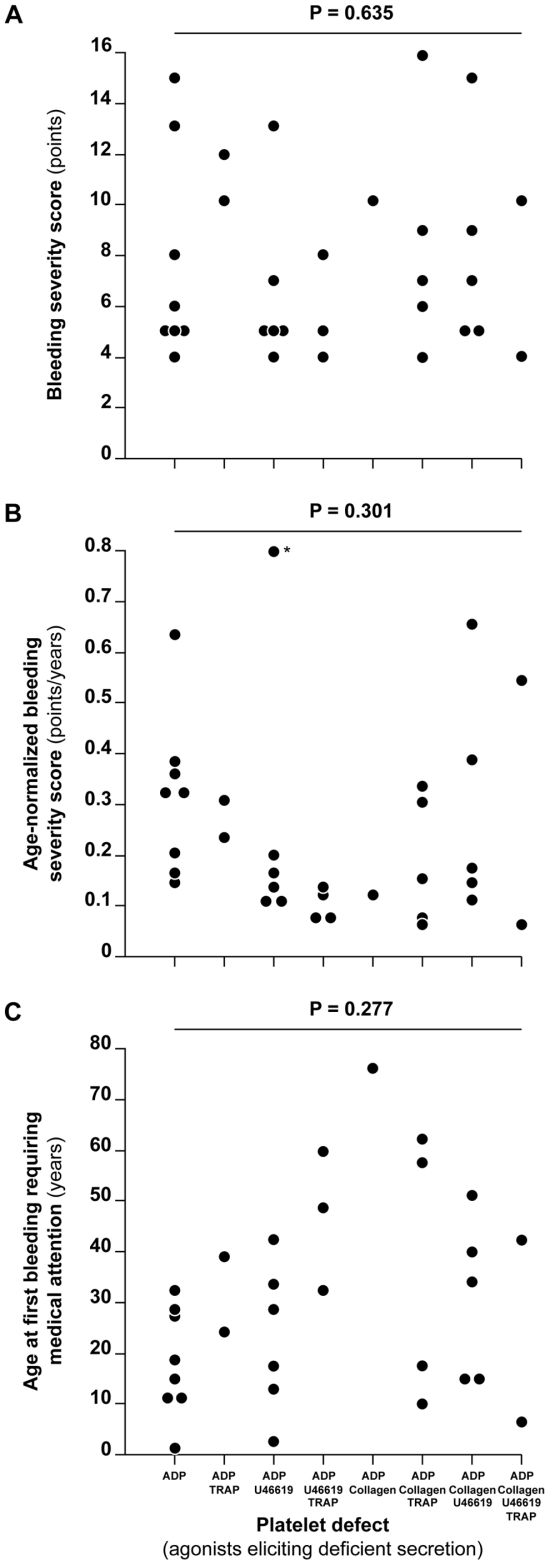
Relationships between measures of bleeding severity and pattern of platelet defect. The Figure shows the distribution of bleeding severity score (top), age-normalized bleeding severity score (middle) and age of first bleeding requiring medical attention (bottom) in patients with different patterns of platelet defect. The asterisk (*) indicates a patient with age-normalized bleeding severity score of 1.89. P-values were calculated by Kruskal-Wallis test.

We sought to determine whether the type and extension of platelet defect was associated with proxies of bleeding severity. The number of agonists eliciting reduced secretion at any concentration or at maximal concentration of the agonist was not associated with BSS, age-normalized BSS or age at first bleeding requiring medical attention at linear regression analysis (Table 3). The lack of association was observed both in an unadjusted analysis and after adjusting for potential confounders (Table 3). No association was found even after stratifying for the presence of associated medical conditions or after excluding patients with defect only upon stimulation with ADP (Tables S4, S5 and S6). We plotted the pattern of platelet defect against BSS, age-normalized BSS and age at first bleeding requiring medical attention (Figure 2A–C). While the values of those proxies of bleeding severity were clustered for some pattern of deficiency (e.g. combined ADP, U46619 and TRAP deficiency), they were spread on a wider range for other deficiencies (e.g. combined ADP, collagen and TRAP deficiency). Overall, the median values of the different proxies of bleeding severity were not different across patterns of platelet defect (Figure 2A–C). No difference in the median was observed also across patterns of platelet defect upon maximal agonist stimulation (pattern vs BSS, P = 0.342; pattern vs age-normalized BSS, P = 0.585; pattern vs age at first bleeding requiring medical attention, P = 0.132; all P-values calculated by Kruskal-Wallis test). No association was found also when investigating only groups of patients with or without accompanying medical conditions (not shown).

**Table 3:** Association between bleeding severity score and platelet secretion testing results in 32 patients with primary secretion defects.

Variable	Bleeding severity score		Age-normalized bleeding severity score		Age of first bleed requiring medical attention	
Type of analysis	Unadjusted	Adjusted^a^	Unadjusted	Adjusted^b^	Unadjusted	Adjusted^b^
Number of agonists with reduced response						
Beta (95% CI)	0.1 (−1.6 to 1.4)	−0.4 (−2.0 to 1.3)	−0.04 (−0.18 to 0.09)	−0.05 (−0.19 to 0.09)	6.2 (−1.3 to 13.6)	5.8 (−2.1 to 13.8)
R^2^	0.0	0.0	0.0	0.1	0.1	0.1
p-value	0.909	0.675	0.512	0.442	0.100	0.144
Number of agonists with reduced response at maximal stimulation						
Beta (95% CI)	0.9 (−0.8 to 2.5)	0.8 (−1.1 to 2.6)	0.07 (−0.08 to 0.21)	0.06 (−0.10 to 0.21)	4.1 (−4.5 to 12.6)	3.4 (−5.8 to 12.5)
R^2^	0.0	0.1	0.0	0.1	0.0	0.1
p-value	0.290	0.397	0.370	0.454	0.337	0.458

a Adjusted for age at referral, sex, clinic of referral, region of residence.

b Adjusted for sex, clinic of referral, region of residence.

CI, confidence intervals.

## Discussion

We collected information on patients referred to our center for bleeding or hemostatic test abnormalities in the last three years in order to study the prevalence, characteristics and determinants of bleeding severity in patients with PSD. Findings of this study were that (a) PSD is found in one fifth of patients with bleeding diathesis; (b) patients with accompanying medical conditions had different characteristics from those without such conditions; (c) the type and extension of platelet defect was not associated with the severity of bleeding.

The prevalence of PSD was found by Quiroga et al. to be 19% in patients with mucocutaneous bleeding [10] and by Philipp to be 36% in women with menorrhagia [11]. However, in the 2010 World Federation of Hemophilia Global Survey (URL: http://www1.wfh.org/publications/files/pdf-1427.pdf), the prevalence of PSD in patients with bleeding from causes other than von Willebrand disease and hemophilia is not taken into consideration. Nonetheless, it is mentioned in the Survey that many countries reported a high number of patients with “platelet defects other than Glanzmann's thrombasthenia and Bernard-Soulier syndrome”. This underestimation probably reflects the incomplete diffusion of platelet testing, required to identify PSD. In this study, focusing the investigation on all patients with any bleeding symptoms and BSS of 4 and above yielded a prevalence of approximately 19%. Our study shows that the prevalence of these defects is considerable also in patients with an important history of bleeding, indicating the importance of PSD as a cause of clinically relevant bleeding at the population level. These results warrant interventions to make the diagnosis of PSD more widely available. This entails the development and diffusion of assays that can be more easily and rapidly performed. In consideration of the fact that many patients with PSD bleed during surgery or other invasive medical procedures, the detection of the defect at an early age could prevent unnecessary bleeding episodes. Screening for ADP-deficiency might be a sensitive strategy, given that ADP deficiency was herein present in all investigated PSD patients, but specificity of the finding of PSD exclusive to stimulation with ADP might be a concern.

Patients without associated medical conditions had earlier age of first bleeding and different platelet functional defect pattern compared to patients with PSD and accompanying medical conditions. This suggests a possible different etiologic and pathogenic mechanisms in the two groups of patients. The early age of onset in patients without associated conditions indicates congenital defects that may be amenable to genetic mapping.

There was no association, in patients with or without accompanying conditions or in the entire group, between the pattern and extension of platelet functional defect and proxies of the severity of bleeding. One of the reasons for these negative results might have been the tiny sample size of the study. However, the firmly negative results and the complete lack of an association suggest that the effect of platelet functional defect, if any, is likely small. This result suggests that characterizing the type and extension of platelet defect might provide little prognostic information on the severity of bleeding, once a diagnosis of PSD is established.

Our study has limitations. Platelet functional testing was not performed in patients with BSS below 4, not enabling the classification of patients with isolated or very mild bleeding with respect to their PSD status. Although this might have blunted the appreciation of the entire spectrum of the bleeding severity of these conditions, it also restricted the analysis to those patients who have clinically relevant disease. A number of patients were not referred for platelet testing, possibly leading to inaccurate prevalence estimation. To circumvent this limitation, we performed multiple imputation to estimate the prevalence of PSD in this subgroup of patients. However, the prevalence of PSD found in this study was remarkably similar to that described by Quiroga et al. in patients with mucocutaneous bleeding. Even after we excluded all patients with defect only upon stimulation by ADP, the estimate of PSD prevalence remained as high as 14%. These findings indicate that the prevalence of PSD in patients with bleeding remains considerable even when using conservative criteria to define this condition. Because patients with thrombocytopenia were not excluded from platelet functional testing, our prevalence estimation might not be representative of the prevalence of PSD in patients with thrombocytopenia. Another possible limitation of the study is that BSS has been validated in von Willebrand disease type 1 and 3 [12], [13]. Its use in other conditions characterized by mild bleeding tendency has been highly recommended but it is still not validated [20], [21]. Although BSS was not the only proxy of diseases severity in our study, we recognize that its application in a disease different from von Willebrand disease might have partially limited our evaluation of disease severity in patients with PSD. Nonetheless, we herein chose to use BSS for a number of reasons. First, the same type of BSS presented in this study has been successfully used in other bleeding conditions different from the ones it was originally conceived for [20]–[22]. BSS has been previously used in PSD and other investigators have suggested its adoption for the assessment of disease severity in PSD [23], [24]. In addition, similarly to von Willebrand disease, PSD is a defect of primary hemostasis, characterized by mild to moderate bleeding symptoms. Finally, a limitation of the study is that sample size was relatively small. However, we were able to collect a well-characterized cohort of patients, in whom testing of platelet function was accurate and complete. The patient number available for this study was sufficient to have rather precise estimations of the prevalence of these conditions. The study was also empowered to detect large difference between study subgroup and strong, clinically-relevant relationships between study measurements and bleeding severity.

In conclusion, PSD was found by this study to be present in approximately one fifth of patients with bleeding diathesis. In patients with PSD, the severity of bleeding manifestations was not associated with the type and extension of the laboratory defect.

## Supporting Information

Table S1 **Questionnaire used to compile bleeding severity score according to Tosetto et al.** J Thromb Haemost 2006; 4: 766–73. Score is assigned for each symptom category; the final bleeding severity score is the sum of all symptom-category scores.
(DOCX)

Table S2 Prevalence calculation after the exclusion of patients with defect of secretion only upon stimulation with ADP.
(DOCX)

Table S3 Characteristics of 32 patients with primary secretion defects according to the presence of associated conditions.
(DOCX)

Table S4 Association between bleeding severity score and platelet secretion testing results in patients with PSD and no associated medical conditions.
(DOCX)

Table S5 Association between bleeding severity score and platelet secretion testing results in patients with PSD and associated medical conditions.
(DOCX)

Table S6 Association between laboratory results and bleeding severity after the exclusion of patients with defect of secretion only upon stimulation with ADP (patients included in the analysis, n = 24).
(DOCX)

## References

[1] Fuse I (1996) Disorders of platelet function. Crit Rev Oncol Hematol 22: 1–25. 8672250 10.1016/1040-8428(94)00167-7

[2] Cattaneo M (2003) Inherited platelet-based bleeding disorders. J Thromb Haemost 1: 1628–1636. 12871299 10.1046/j.1538-7836.2003.00266.x

[3] Nurden A, Nurden P (2011) Advances in our understanding of the molecular basis of disorders of platelet function. J Thromb Haemost 9 Suppl 1: 76–91. 21781244 10.1111/j.1538-7836.2011.04274.x

[4] Hayward CP (2011) Diagnostic evaluation of platelet function disorders. Blood Rev 25: 169–173. 21497967 10.1016/j.blre.2011.03.004

[5] Rao AK, Jalagadugula G, Sun L (2004) Inherited defects in platelet signaling mechanisms. Semin Thromb Hemost 30: 525–535. 15497095 10.1055/s-2004-835673

[6] Fuse I, Hattori A, Higashihara M, Takizawa S, Takeshige T, et al (1986) A defect of platelet release reaction in a patient with SLE: impaired platelet aggregation induced by phorbol ester with a normal phosphorylation of 40K protein. Scand J Haematol 36: 44–54. 3081995 10.1111/j.1600-0609.1986.tb02648.x

[7] Stuart MJ, Kelton JG, Allen JB (1981) Abnormal platelet function and arachidonate metabolism in chronic idiopathic thrombocytopenic purpura. Blood 58: 326–329. 6788109

[8] Hillbom M, Muuronen A, Neiman J (1987) Liver disease and platelet function in alcoholics. Br Med J (Clin Res Ed) 295: 581. 10.1136/bmj.295.6598.581 PMC1248749 3117241

[9] Cowan DH, Graham RC Jr, Baunach D (1975) The platelet defect in leukemia. Platelet ultrastructure, adenine nucleotide metabolism, and the release reaction. J Clin Invest 56: 188–200. 45818 10.1172/JCI108067 PMC436569

[10] Quiroga T, Goycoolea M, Panes O, Aranda E, Martinez C, et al (2007) High prevalence of bleeders of unknown cause among patients with inherited mucocutaneous bleeding. A prospective study of 280 patients and 299 controls. Haematologica 92: 357–365. 17339185 10.3324/haematol.10816

[11] Philipp CS, Dilley A, Miller CH, Evatt B, Baranwal A, et al (2003) Platelet functional defects in women with unexplained menorrhagia. J Thromb Haemost 1: 477–484. 12871453 10.1046/j.1538-7836.2003.00061.x

[12] Castaman G, Rodeghiero F, Tosetto A, Cappelletti A, Baudo F, et al (2006) Hemorrhagic symptoms and bleeding risk in obligatory carriers of type 3 von Willebrand disease: an international, multicenter study. J Thromb Haemost 4: 2164–2169. 16999850 10.1111/j.1538-7836.2006.02070.x

[13] Tosetto A, Rodeghiero F, Castaman G, Goodeve A, Federici AB, et al (2006) A quantitative analysis of bleeding symptoms in type 1 von Willebrand disease: results from a multicenter European study (MCMDM-1 VWD). J Thromb Haemost 4: 766–773. 16634745 10.1111/j.1538-7836.2006.01847.x

[14] Lotta LA, Lombardi R, Mariani M, Lancellotti S, De Cristofaro R, et al (2011) Platelet reactive conformation and multimeric pattern of von Willebrand factor in acquired thrombotic thrombocytopenic purpura during acute disease and remission. J Thromb Haemost 9: 1744–1751. 21726405 10.1111/j.1538-7836.2011.04428.x

[15] Bowman M, Mundell G, Grabell J, Hopman WM, Rapson D, et al (2008) Generation and validation of the Condensed MCMDM-1VWD Bleeding Questionnaire for von Willebrand disease. J Thromb Haemost 6: 2062–2066. 18983516 10.1111/j.1538-7836.2008.03182.x

[16] Mannucci PM, Cattaneo M, Canciani MT, Maniezzo M, Vaglini M, et al (1989) Early presence of activated ('exhausted') platelets in malignant tumors (breast adenocarcinoma and malignant melanoma). Eur J Cancer Clin Oncol 25: 1413–1417. 2531669 10.1016/0277-5379(89)90098-9

[17] Cattaneo M (2009) Light transmission aggregometry and ATP release for the diagnostic assessment of platelet function. Semin Thromb Hemost 35: 158–167. 19408189 10.1055/s-0029-1220324

[18] Cattaneo M, Lecchi A, Lombardi R, Gachet C, Zighetti ML (2000) Platelets from a patient heterozygous for the defect of P2CYC receptors for ADP have a secretion defect despite normal thromboxane A2 production and normal granule stores: further evidence that some cases of platelet 'primary secretion defect' are heterozygous for a defect of P2CYC receptors. Arterioscler Thromb Vasc Biol 20: E101–106. 11073862 10.1161/01.atv.20.11.e101

[19] Agresti A, Coull BA (1998) Approximate is better than "exact" for interval estimation of binomial proportions. American Statistician 52: 119–126.

[20] Rodeghiero F, Tosetto A, Abshire T, Arnold DM, Coller B, et al (2010) ISTH/SSC bleeding assessment tool: a standardized questionnaire and a proposal for a new bleeding score for inherited bleeding disorders. J Thromb Haemost 8: 2063–2065. 20626619 10.1111/j.1538-7836.2010.03975.x

[21] Tosetto A, Castaman G, Plug I, Rodeghiero F, Eikenboom J (2011) Prospective evaluation of the clinical utility of quantitative bleeding severity assessment in patients referred for hemostatic evaluation. J Thromb Haemost 9: 1143–1148. 21435168 10.1111/j.1538-7836.2011.04265.x

[22] O'Brien SH (2012) Bleeding scores: are they really useful? Hematology Am Soc Hematol Educ Program 2012: 152–156. 23233574 10.1182/asheducation-2012.1.152

[23] Podda G, Femia EA, Pugliano M, Cattaneo M (2012) Congenital defects of platelet function. Platelets 23: 552–563. 23020613 10.3109/09537104.2012.724737

[24] Cattaneo M (2011) Molecular defects of the platelet P2 receptors. Purinergic Signal 7: 333–339. 21484091 10.1007/s11302-011-9217-z PMC3166989

