# Supplementary material for: Prevalence of Disease and Relationships between Laboratory Phenotype and Bleeding Severity in Platelet Primary Secretion Defects
Source: PLoS One. 2013 Apr 2;8(4):e60396. doi: 10.1371/journal.pone.0060396 (PMC3614926; doi:10.1371/journal.pone.0060396)
Supplement: Table S1 — Questionnaire used to compile bleeding severity score according to Tosetto et al. J Thromb Haemost 2006; 4: 766–73. Score is assigned for each symptom category; the final bleeding severity score is the sum of all symptom-category scores. (DOCX) [file pone.0060396.s001.docx]

**Table S1**

|  | **Score** | | | | | |
| --- | --- | --- | --- | --- | --- | --- |
| **Symptom** | -1 | 0 | 1 | 2 | 3 | 4 |
| **Epistaxis** | / | No or trivial  (less than 5) | >5 or more than 10 | Consultation only | Packing or cauterization  or anti-ﬁbrinolytic drug | Blood transfusion or replacement therapy or desmopressin |
| **Cutaneous** | / | No or trivial  (<1 cm) | >1 cm and no trauma | Consultation only |  |  |
| **Bleeding from minor wounds** | / | No or trivial  (less than 5) | >5 or more than 5’ | Consultation only | Surgical hemostasis | Blood transfusion  or replacement  therapy or  desmopressin |
| **Oral cavity** | / | No | Referred at least one | Consultation only | Surgical hemostasis  or anti-fibrinolytic drug | Blood transfusion  or replacement  therapy or  desmopressin |
| **Gastrointestinal bleeding** | / | No | Associated with ulcer,  portal hypertension,  hemorrhoids,  angiodysplasia | Spontaneous | Surgical hemostasis,  blood transfusion,  replacement therapy,  desmopressin,  anti-fibrinolytic drug | / |
| **Tooth extraction** | No bleeding  in at least  two extraction | None done or  no bleeding in  one extraction | Referred in <25%  of all procedures | Referred in >25%  of all procedures,  no intervention | Resuturing or  packing | Blood transfusion  or replacement  therapy or  desmopressin |
| **Surgery** | No bleeding  in at least  two surgeries | None done or  no bleeding in  one surgery | Referred in <25%  of all surgeries | Referred in >25%  of all procedures,  no intervention | Surgical hemostasis  or anti-fibrinolytic | Blood transfusion  or replacement  therapy or  desmopressin |
| **Menorrhagia** | / | No | Consultation only | Anti-fibrinolytic drug, pill use | Dilatation and  curettage, iron  therapy | Blood transfusion  or replacement  therapy or  desmopressin or  hysterectomy |
| **Postpartum hemorrhage** | No bleeding  in at least  two deliveries | No deliveries or  no bleeding in  one delivery | Consultation only | Dilatation and curettage,  iron therapy,  anti-fibrinolytic drug | Blood transfusion or  replacement therapy  or desmopressin | Hysterectomy |
| **Muscle hematomas** | / | Never | Post trauma no  therapy | Spontaneous, no therapy | Spontaneous or  traumatic, requiring  desmopressin or  replacement therapy | Spontaneous or  traumatic,  requiring surgical  intervention or  blood transfusion |
| **Hemarthrosis** | / | Never | Post trauma no  therapy | Spontaneous, no therapy | Spontaneous or  traumatic, requiring  desmopressin or  replacement therapy | Spontaneous or  traumatic,  requiring Surgical  intervention or  blood transfusion |
| **Central nervous**  **system bleeding** | / | Never | / | / | Subdural, any  intervention | Intracerebral,  any intervention |
