# Supplementary material for: Prevalence of Disease and Relationships between Laboratory Phenotype and Bleeding Severity in Platelet Primary Secretion Defects
Source: PLoS One. 2013 Apr 2;8(4):e60396. doi: 10.1371/journal.pone.0060396 (PMC3614926; doi:10.1371/journal.pone.0060396)
Supplement: Table S2 — Prevalence calculation after the exclusion of patients with defect of secretion only upon stimulation with ADP. (DOCX) [file pone.0060396.s002.docx]

**Table S2**

| **Patient group** | **N =** | **Prevalence of PSD, % (n=)** | **95% confidence intervals of prevalence estimation^c^, %** |
| --- | --- | --- | --- |
| Referred for platelet testing | 145 | 14.5 (21) | 9.5-18.9 |
| Not referred^a^ | 62 | 11.4 (7)^b^ | 5.3-21.8 |
| All | 207 | 13.5 (28) | 9.6-21.2 |

a Estimated by multiple imputation

b Based on an imputed count of 7 patients with PSD in this group

c Calculated according to Agresti-Coull

PSD: primary secretion defect
