# Supplementary material for: Prevalence of Disease and Relationships between Laboratory Phenotype and Bleeding Severity in Platelet Primary Secretion Defects
Source: PLoS One. 2013 Apr 2;8(4):e60396. doi: 10.1371/journal.pone.0060396 (PMC3614926; doi:10.1371/journal.pone.0060396)
Supplement: Table S3 — Characteristics of 32 patients with primary secretion defects according to the presence of associated conditions. (DOCX) [file pone.0060396.s003.docx]

| **Variable** | **PSD without associated conditions (n=22)** | **PSD with associated conditions (n=10)** | **Difference**  **(95% CI)** |
| --- | --- | --- | --- |
| Median age at referral, y (IQR) | 34  (18-43) | 50  (40-63) | -16  (-34 to 1) |
| Median age at first bleeding requiring medical attention, y (IQR) | 18  (12-33) | 45  (35-60) | -27  (-46 to -9) |
| Female sex, n (%) | 17 (77) | 7 (70) | 7  (-2 to 40) |
| Median bleeding severity score, points (IQR) | 6.5 (5-9) | 7.5 (4-10.5) | -1  (-4.7 to 2.73) |
| Median age-adjusted bleeding score, points/y (IQR) | 0.20  (0.15-0.37) | 0.13  (0.08-0.26) | 0.08  (-0.06 to 0.22) |
| Secretion defect upon stimulation^a^ |  | | |
| ADP any concentration, n (%) | 22 (100) | 10 (100) | 0 (-15 to 28) |
| ADP 20 μM, n (%) | 15 (68) | 9 (90) | -22 (-44 to 12) |
| Collagen any concentration, n (%) | 6 (27) | 7 (70) | -43 (-67 to 6) |
| Collagen 20 μg/mL, n (%) | 1 (5) | 0 | 5 (-24 to 22) |
| U46619 any concentration, n (%) | 11(50) | 5 (50) | 0 (-33 to 33) |
| U46619 1 μM, n (%) | 6 (27) | 4 (40) | -13 (-45 to 19) |
| TRAP any concentration, n (%) | 6 (27) | 6 (60) | -33 (-60 to 3) |
| TRAP 20 μM, n (%) | 1 (5) | 2 (20) | -15 (-47 to 1) |
| Number of agonists with reduced response, n (%) |  | | |
| 1 agonist | 8 (36) | 0 | 36 (4 to 57) |
| 2 agonists | 6 (27) | 3 (30) | -3 (-36 to 3) |
| 3 agonists | 7 (32) | 6 (60) | -28(-56 to 7) |
| 4 agonists | 1 (5) | 1 (10) | -5 (-36 to 1) |
| Number of agonists with reduced response at maximal stimulation, n (%) |  |  |  |
| 0 agonists | 6 (27) | 1(10) | 17 (-2 to 40) |
| 1 agonist | 10 (46) | 3(30) | 16 (-2 to 43) |
| 2 agonists | 5 (23) | 6 (60) | -37(-63 to 2) |
| 3 agonists | 1 (5) | 0 | 5 (-24 to 22) |
| Pattern of platelet defect, n (%) |  | | |
| ADP | 8 (36) | 0 | 36 (4 to 57) |
| ADP, TRAP | 0 | 2 (20) | -20 (-51 to 1) |
| ADP, U46619 | 6 (27) | 0 | 27 (-4 to 48) |
| ADP, U46619, TRAP | 2 (9) | 1 (10) | -1 (-32 to 20) |
| ADP, collagen | 0 | 1 (10) | -10 (-40 to 7) |
| ADP, collagen, TRAP | 3 (14) | 2 (20) | -6 (-39 to 18) |
| ADP, collagen, U46619 | 2 (9) | 3 (30) | -21 (-52 to 6) |
| ADP, collagen, U46619, TRAP | 1 (5) | 1 (10) | -5 (-36 to 14) |

**Table S3**

a Number and percentage of patients showing reduced ATP secretion upon stimulation by the reported agonist at the reported concentration.

IQR, interquartile range; ADP, adenosine diphosphate; TRAP, thrombin receptor-activating peptide.
