# Supplementary material for: Prevalence of Disease and Relationships between Laboratory Phenotype and Bleeding Severity in Platelet Primary Secretion Defects
Source: PLoS One. 2013 Apr 2;8(4):e60396. doi: 10.1371/journal.pone.0060396 (PMC3614926; doi:10.1371/journal.pone.0060396)
Supplement: Table S4 — Association between bleeding severity score and platelet secretion testing results in patients with PSD and no associated medical conditions. (DOCX) [file pone.0060396.s004.docx]

**Table S4**

| **Parameter** | **Bleeding severity score** | | **Age-normalized bleeding severity score** | | **Age of first bleed requiring medical attention** | |
| --- | --- | --- | --- | --- | --- | --- |
| **Type of analysis** | Unadjusted | Adjusted^a^ | Unadjusted | Adjusted^b^ | Unadjusted | Adjusted^b^ |
| **Number of agonists with reduced response** |  |  |  |  |  |  |
| Beta (95% CI) | 0.4  (-1.3 to 2.2) | 0.1  (-1.8 to 2.1) | -0.01  (-0.19 to 0.18) | -0.04  (-0.24 to 0.17) | 2.0  (-5.2 to 9.2) | 2.6  (-5.8 to 10.9) |
| R^2^ | 0.1 | 0.2 | 0.0 | 0.1 | 0.0 | 0.0 |
| p-value | 0.606 | 0.888 | 0.910 | 0.712 | 0.567 | 0.526 |
| **Number of agonists with reduced response at maximal stimulation** |  |  |  |  |  |  |
| Beta (95% CI) | 0.8  (-1.2 to 2.7) | 0.5  (-1.6 to 2.6) | 0.01  (-0.10 to 0.30) | 0.1  (-0.13 to 0.32) | 1.5  (-6.5 to 9.4) | 1.5  (-7.7 to 10.6) |
| R^2^ | 0.0 | 0.2 | 0.1 | 0.1 | 0.1 | 0.0 |
| p-value | 0.429 | 0.609 | 0.300 | 0.381 | 0.707 | 0.733 |

a Adjusted for age at referral, sex, clinic of referral, region of residence

b Adjusted for sex, clinic of referral, region of residence
