# Supplementary material for: Prevalence of Disease and Relationships between Laboratory Phenotype and Bleeding Severity in Platelet Primary Secretion Defects
Source: PLoS One. 2013 Apr 2;8(4):e60396. doi: 10.1371/journal.pone.0060396 (PMC3614926; doi:10.1371/journal.pone.0060396)
Supplement: Table S5 — Association between bleeding severity score and platelet secretion testing results in patients with PSD and associated medical conditions. (DOCX) [file pone.0060396.s005.docx]

**Table S5**

| **Parameter** | **Bleeding severity score** | | **Age-normalized bleeding severity score** | | **Age of first bleed requiring medical attention** | |
| --- | --- | --- | --- | --- | --- | --- |
| **Type of analysis** | Unadjusted | Adjusted^a^ | Unadjusted | Adjusted^b^ | Unadjusted | Adjusted^b^ |
| **Number of agonists with reduced response** |  |  |  |  |  |  |
| Beta (95% CI) | -3.4  (-7.6 to 0.8) | -6.2  (-12.7 to 0.3) | -0.06  (-0.30 to 0.17) | -0.26  (-0.57 to 0.06) | -1.5  (-24.5 to 21.6) | 6.9  (-32.9 to 46.6) |
| R^2^ | 0.3 | 0.8 | 0.0 | 0.7 | 0.0 | 0.4 |
| p-value | 0.097 | 0.057 | 0.572 | 0.089 | 0.886 | 0.676 |
| **Number of agonists with reduced response at maximal stimulation** |  |  |  |  |  |  |
| Beta (95% CI) | 1.2  (-3.1 to 5.6) | 0.0  (-7.6 to 7.7) | -0.06  (-0.14 to 0.27) | -0.03  (-0.33 to 0.27) | -2.2  (-22.7 to 18.4) | 8.9  (-17.7 to 35.4) |
| R^2^ | 0.1 | 0.4 | 0.1 | 0.348 | 0.1 | 0.5 |
| p-value | 0.535 | 0.995 | 0.498 | 0.809 | 0.813 | 0.430 |

a Adjusted for age at referral, sex, clinic of referral, region of residence

b Adjusted for sex, clinic of referral, region of residence
