# Supplementary material for: Prevalence of Disease and Relationships between Laboratory Phenotype and Bleeding Severity in Platelet Primary Secretion Defects
Source: PLoS One. 2013 Apr 2;8(4):e60396. doi: 10.1371/journal.pone.0060396 (PMC3614926; doi:10.1371/journal.pone.0060396)
Supplement: Table S6 — Association between laboratory results and bleeding severity after the exclusion of patients with defect of secretion only upon stimulation with ADP (patients included in the analysis, n = 24). (DOCX) [file pone.0060396.s006.docx]

**Table S6**

| **Variable** | **Bleeding severity score** | | **Age-normalized bleeding severity score** | | **Age of first bleed requiring medical attention** | |
| --- | --- | --- | --- | --- | --- | --- |
| **Type of analysis** | Unadjusted | Adjusted^a^ | Unadjusted | Adjusted^b^ | Unadjusted | Adjusted^b^ |
| **Number of agonists with reduced response** |  | | | | | |
| Beta (95% CI) | -0.34  (-2.85 to 2.17) | -0.79  (-3.79 to 2.21) | -0.08  (-0.34 to 0.18) | -0.12  (-0.42 to 0.18) | 1.3  (-12.7 to 15.4) | 4.9  (-11.1 to 21.1) |
| R^2^ | 0.1 | 0.1 | 0.1 | 0.1 | 0.1 | 0.1 |
| p-value | 0.782 | 0.587 | 0.527 | 0.423 | 0.846 | 0.523 |

a Adjusted for age at referral, sex, clinic of referral, region of residence

b Adjusted for sex, clinic of referral, region of residence
